# Comparison of T cell response to vaccination in rheumatic patients treated with Janus kinase inhibitors and TNF inhibitors

**DOI:** 10.1186/s41927-025-00542-7

**Published:** 2025-07-09

**Authors:** Sebastian Hüper, Florian Eisele, Johannes Duell, Marc Schmalzing, Lea Nagler, Patrick Pascal Strunz, Matthias Froehlich, Jan Portegys, Michael Gernert

**Affiliations:** 1 Department of Internal Medicine II, Rheumatology/Clinical Immunology, University Hospital Würzburg, Oberdürrbacher Straße 6, 97080 Würzburg, Germany; 2 Practice for Rheumatology and Osteology, Bahnhofsplatz 5, 31134 Hildesheim, Germany; 3 Department of Internal Medicine II, Haematology and Oncology, University Hospital Würzburg, Oberdürrbacher Straße 6, 97080 Würzburg, Germany

**Keywords:** Janus kinase inhibitors, Vaccination, T cell response, SARS-CoV-2 vaccination, JAK- inhibitors, TH1-response, TNF- inhibitors

## Abstract

**Background:**

Janus kinase inhibitors (JAKi) represent a well-established therapeutic option for the treatment of autoimmune diseases. However, there is a paucity of evidence regarding their impact on de novo immune responses to vaccinations. T cells may confer long-lasting immunity and cross-recognise evolving epitopes of new viral variants, as evidenced by the SARS-CoV-2 vaccination. Consequently, we investigated the de novo T-cell response to SARS-CoV-2 vaccination in patients with rheumatic diseases undergoing treatment with JAK inhibitors.

**Methods:**

Cross-sectional study, conducted in an outpatient department. Patients with rheumatic disease who had received two vaccinations against SARS-CoV-2 while under therapy with JAKi (n = 22) or tumour necrosis factor-blocking biologicals (TNFi) (control group n = 16) were recruited. To evaluate the vaccine-induced T cell response, the patients’ PBMCs were stimulated with SARS-CoV-2 spike protein peptides. The percentage of CD4^+^ T cells responding specifically to this stimulation by producing IFNγ was then measured using intracellular cytokine staining and flow cytometry. In addition antibody response to vaccination was assessed.

**Results:**

A specific T cell response was detected in 11 out of 22 (50.0%) of patients in the JAKi cohort, compared to 13 out of 16 (81.3%) of the TNFi cohort (*p* = 0.088). Patients on JAKi had a lower percentage of CD4^+^ T cells responding to stimulation with SARS-CoV-2 spike peptides than patients on TNFi (*p* = 0.021). The proportion of patients with an antibody response and absolute anti-spike IgG levels did not significantly differ between the cohorts.

**Conclusions:**

Patients on JAKi exhibited a compromised de novo T cell response to SARS-CoV-2 vaccination compared to TNFi patients. There is a need for further research on the effect of JAKi on T cell responses to vaccination.

**Supplementary Information:**

The online version contains supplementary material available at 10.1186/s41927-025-00542-7.

## Background

In the past decade, Janus kinase inhibitors (JAKi) have been approved as a new class of immunosuppressants. They have become firmly established in treatment algorithms due to their oral administration and efficacy [1]. Studies on the immune response to vaccination under JAKi therapy indicate a significant reduction in antibody response [2–5]. In contrast, there are fewer studies that measure T cell responses, and the results are inconsistent [4, 6, 7]. To improve response to vaccination, temporarily pausing JAKi is an option if the disease is well controlled, but evidence for this approach is scarce [8].

There are indications that JAKi impair the T-cell responses to new vaccine antigens. One reason is their mechanism of action. Janus kinases are present in T and B cells, as well as in cells of the innate immune response, such as antigen-presenting dendritic cells and NK cells. JAKi affect all these cells, but the activation of the downstream signalling cascade is not continuously inhibited due to the short half-life of JAKi [9]. This results in a limited ability of dendritic cells to stimulate T cells and impaired T cell proliferation and Th1-responses [10].

Another argument for impaired T cell function in JAKi patients is the indication of an increased rate of infections compared to TNFi patients. This was demonstrated in the ORAL Surveillance study for tofacitinib, which showed an increased rate of serious infections [11]. While an increased incidence of herpes zoster is well characterised, opportunistic infections associated with very severe T cell dysfunction, such as Pneumocystis jirovecii pneumonia or aspergillosis, have rarely been reported in JAKi patients taking approved doses [12].

During the SARS-CoV-2 pandemic, new vaccines became available which confer protection against severe disease. In immunocompetent individuals, this protection is achieved by strong vaccine-elicited B- and T-cell responses to the virus’s epitopes [13]. In the post-approval phase a large number of studies investigating the immune response to vaccination in patients on immunosuppressive drugs have been published [4, 6, 14, 15].

The objective of this study was to further examine T cell responses to vaccination in patients with rheumatic diseases on JAKi therapy. The vaccination against SARS-CoV-2 in patients whom had not been infected before, provided a unique opportunity to study de novo T cell responses. Patients receiving tumour necrosis factor-α-targeting biologics (TNFi) were chosen as the control group. This was because (a) these patients were likely to have the same underlying diseases and concomitant medications, (b) there was evidence from other vaccinations and early data on SARS-CoV-2 vaccinations that TNFi do not hamper vaccination success [15, 16].

Based on the aforementioned considerations, we aimed to investigate whether patients with rheumatic diseases taking JAK inhibitors exhibit impaired SARS-CoV2-specific T cell and IgG vaccination responses compared to patients taking TNFi.

## Methods

### Study design

This cross-sectional study was conducted at the Rheumatology Clinic of the University Hospital Würzburg between August 2021 and January 2022. Approval for the study was received from the local ethics committee of the University of Würzburg.

The study recruited patients diagnosed with rheumatoid arthritis (RA) or spondylarthritis, including axial spondylarthritis and psoriatic arthritis. The JAKi cohort consisted of patients on therapy with a JAKi, (baricitinib, upadacitinib, tofacitinib, or filgotinib). The control cohort included patients treated with a TNFi, (infliximab, adalimumab, golimumab, certolizumab, or etanercept). To be included, patients had to have received two vaccinations against SARS-CoV-2, with the most recent vaccination being at least 10 days ago. The vaccines available at the time were BNT162b2 (Tozinameran; Pfizer-BioNTech), CX-024414 (Elasomeran; Moderna), or ChAdOx1 nCoV-19 (AstraZeneca). In accordance with the national recommendations at the time of the study’s commencement, two doses of any of the aforementioned vaccines were deemed to constitute the baseline level of immunization. Patients who were concurrently taking immunosuppressive medications other than methotrexate (MTX) or corticosteroids in excess of 15 mg prednisolone-equivalent were excluded. A history of infection with SARS-CoV-2 constituted an exclusion criterion.

### Measurement of cellular immunity and antibody response

To measure the specific T cell response to vaccination, patient samples were stimulated with SARS-CoV-2 spike protein peptides and an intracellular cytokine staining (ICS) for IFNγ in CD4^+^ T cells was subsequently performed.

In brief, peripheral blood mononuclear cells (PBMCs) were isolated from whole blood by density gradient centrifugation using Biocoll (Biochrom, Berlin, Germany). The PBMCs were then stimulated for three hours with either PMA/ionomycin (positive control), medium (negative control), or a SARS-CoV-2 spike protein peptide library (PepTivator SARS-CoV-2 Prot S Complete, Miltenyi Biotec, Bergisch Gladbach, Germany). Brefeldin A was then added to block cytokine secretion. The cells were then fixed using IC Fix/Perm buffer (Sigma-Aldrich, USA) and stained with CD3-PerCP, CD4-BV510 and IFNγ-FITC (all BioLegend, UK). Analysis was performed immediately using a BD FACS Canto II flow cytometer (BD Biosciences, USA). The data were analysed using Kaluza Analysis Software 1.3 (Beckman Coulter, Brea, USA) (see Additional File 1 for the gating strategy). ICS results were analysed as described by Liu et al. [17]. To account for pre-existing and non-spike-specific IFNγ^+^ cells, the percentage of IFNγ^+^/CD4^+^ T cells in the spike-stimulated samples was adjusted by subtracting the percentage of the respective unstimulated negative control. Any negative values were set to zero. To compare the magnitude of the specific T cell responses between the groups, the median frequency of IFNγ^+^/CD4^+^ T cells following stimulation was calculated.

Anti-spike IgG (anti-S IgG) was determined by chemiluminescence immunoassay (CLIA) (LIAISON^®^ SARS-CoV-2 TrimericS IgG, Diasorin, Saluggia, Italy). Serological response was defined by the binding antibody unit (BAU) cut-off according to the manufacturer’s instructions (> 34 BAU/ml). In parallel, testing for antibodies against the nucleocapsid protein (anti-N IgG), indicative for previous infection with SARS-CoV-2, was performed using an electrochemiluminescence immunoassay (Elecsys^®^ Anti-SARS-CoV-2 N-Assay, Roche, Basel, Switzerland).

### Data analysis and statistics

Statistical analysis was conducted using GraphPad Prism Software (San Diego, USA). An unpaired t-test with Welch’s correction was used to compare mean values. The mean values and the corresponding interquartile range (IQR) were reported. Mann-Whitney test was used to compare median responses and to obtain exact two-tailed P values. Response rates were compared using a two-tailed Fisher’s exact test. Spearman’s rank correlation coefficient was used to test for correlations. Graphs were created using CorelDraw Software (Corel Corporation, Ottawa, Canada).

## Results

### Patient numbers and characteristics

22 patients were included in the JAKi cohort and 16 patients in the TNFi cohort. Although the demographic characteristics, such as age and sex, were similar, the two cohorts differed significantly in terms of underlying diseases and immunosuppressive co-medications. All patients in the JAKi cohort had RA, whereas half of the TNFi cohort had spondylarthritis. Approximately one third of patients in the JAKi cohort took corticosteroids; none in the TNFi group did. The mean lymphocyte count was significantly higher in the TNFi cohort (*p* = 0.0001) (Table 1).

**Table 1:** Patients characteristics

	JAKi cohort (*n* = 22)	TNFi cohort (*n* = 16)	*P* value
Age, mean years ± SD	64.5 ± 6.4	64.2 ± 6.2	0.920
Female, %	63.6	56.2	0.742
Male, %	36.4	43.8	
Rheumatoid arthritis, n, %	22/22 (100.0%)	8/16 (50.0%)	< 0.001
Spondylarthritis, n, %	0/22 (0.0%)	8/16 (50.0%)	
Patients with rheumatoid arthritis positive for anti-CCP antibodies, n, %	18/22 (81.7%)	7/8 (87.5)	n.a.
Disease duration, mean years ± SD	15.3 ± 6.8	22.5 ± 8.0	0.050
Methotrexate intake, n, %	7/22 (32.0%)	8/16 (50.0%)	0.324
Average methotrexate dose/week of the patients with intake, mg ± SD	13.9 ± 3.0	11.3 ± 2.5	n.a.
Corticosteroid intake, %	8/22(36.0%)	0/16 (0.0%)	0.012
Average daily dose of the patients with intake, prednisolone-equivalent in mg	4.8	0.0	n.a
Lymphocytes, mean count/µl ± SD	1524 ± 449	2405 ± 473	< 0.001
Mean duration between 2nd vaccination and sampling, days ± SD	139.9 ± 35.9	145.5 ± 19.8	0.691
Pausing medication around vaccinations, n, %	5/22 (23.0%)	5/16 (31.3%)	> 0.999
	**Type of JAKi**	**Type of TNFi**	n.a
	Baricitinib: 13/22 (59.0%)	Adalimumab: 8/16 (50.0%)	
	Tofacitinib: 1/22(5.0%)	Infliximab: 1/16 (6.3%)	
	Upadacitinib: 7/22 (32.0%)	Golimumab: 3/16 (18.8%)	
	Filgotinib: 1/22 (5.0%)	Etanercept: 4/16 (25.0%)	

n.a- not applicable; SD- standard deviation; P values were calculated with an unpaired t-test with Welch’s correction

### CD4^+^ T cell response to vaccination

**Fig. 1:**
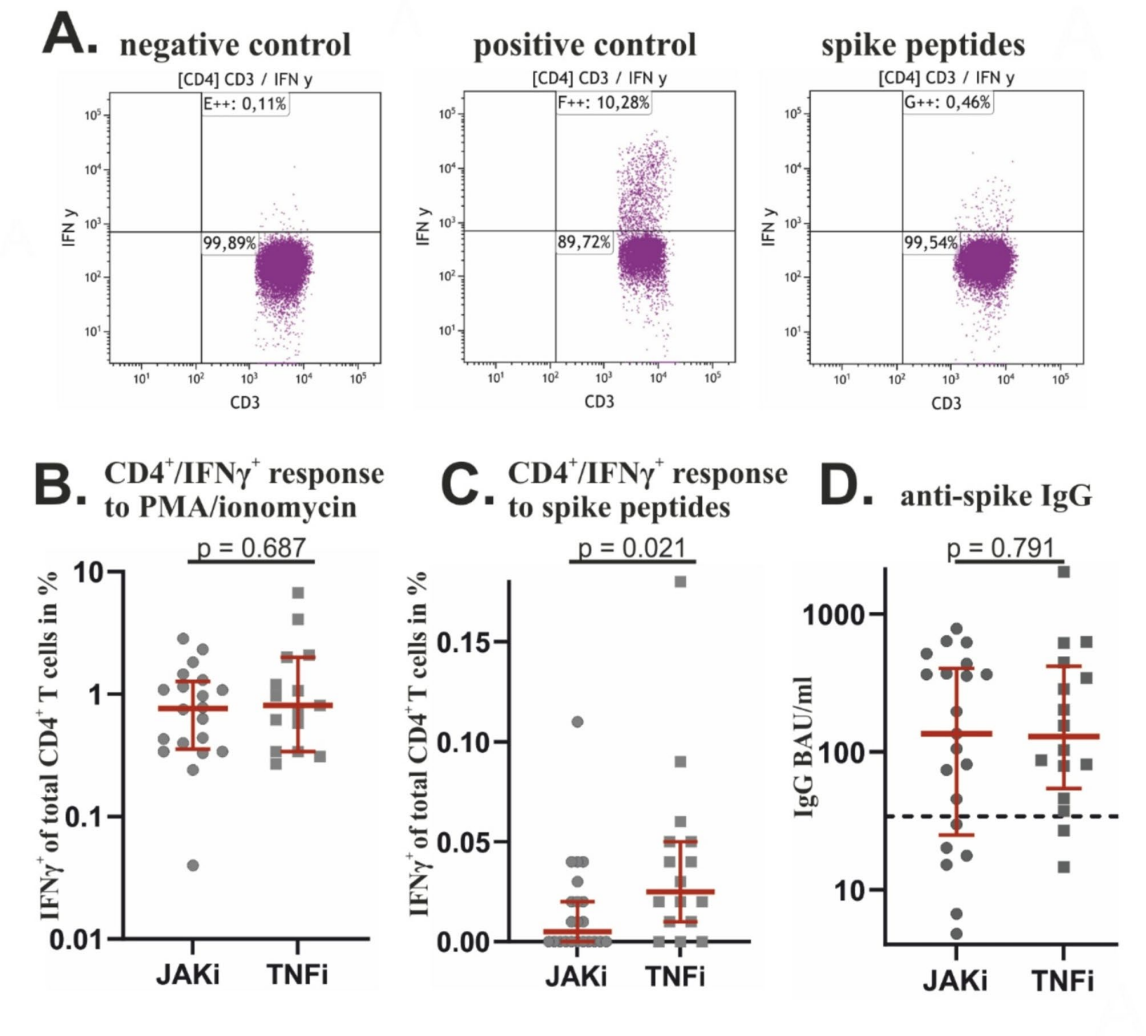
CD4^+^ T cell and IgG responses to SARS-CoV-2 spike protein upon vaccination. **A**. Example of ICS dot plots after gating on CD4^+^ T cells from a patient on TNFi. During stimulation period PBMCs were incubated either with medium (negative control), PMA/ionomycin (positive control), or SARS-CoV-2 spike-protein peptide library (spike peptides) **B**. Frequency of IFNγ^+^/CD4^+^ T cells after non-specific stimulation with PMA/ionomycin. **C**. Frequency of IFNγ^+^/CD4^+^ T cells after stimulation with SARS-CoV-2 peptide pool. **D**. Serum levels of anti-spike IgG. The dotted line indicates the cut-off for positivity (34 BAU/ml). In **B**., **C**. and **D**. each dots represents one patient. In B. and C., correction for pre-existing IFNγ^+^/CD4^+^ T cells was performed by subtraction of the respective negative control. The median values and the corresponding IQR are indicated. To compare the median values, a two-sided Mann-Whitney test was performed

Upon stimulation and ICS flow cytometry was performed to asses IFNγ production in CD4^+^ T cells (Fig. 1A).

For positive controls samples had been stimulated with PMA/ionomycin. Following stimulation with PMA/ionomycin, the median frequency of IFNγ^+^/CD4^+^ T cells did not differ significantly between the JAKi cohort (0.765%, IQR 0.355–1.270%) and the TNFi cohort (0.810%, IQR 0.340–2.000%) (*p* = 0.687) (Fig. 1B). Thus, the ability of CD4^+^ T cells to produce IFNγ in response to non-specific stimulation was similar in both cohorts.

In regard to the response to vaccination 11out of 22 (50%) patients in the JAKi cohort, compared to 13 out of 16 (81.3%) patients in the TNFi cohort, showed an S-peptide specific IFNγ response in CD4^+^ T cells. However, this difference in response rate was not statistically significant (*p* = 0.088). The strength of the T cell response to vaccination, as measured by the median frequency of specific CD4^+^ T cells, was found to be weaker in the JAKi cohort (0.005%, IQR 0.000–0.023%) than in the TNFi cohort (0.025%, IQR 0.010–0.050%) (*p* = 0.021) (Fig. 1C).

A slight correlation was observed between the patients’ lymphocyte count and the frequency of SARS-CoV-2-specific T cells, although this did not reach statistical significance. Furthermore, no correlation was found between patient age or the time elapsed between the second vaccination and sample collection and the patient’s T cell responses. The median frequency of specific CD4^+^/IFNγ^+^ T cells did not differ significantly between patients with and without concomitant therapy with methotrexate, or glucocorticoid use, or those who temporarily paused immunosuppressive medication and those who did not (Additional file 2).

### Antibody response to vaccination

Except for one patient in the JAKi cohort, antibody testing for anti-S IgG and for anti-N IgG was available. None of the patients had anti-N IgG as a serological marker of previous infection with SARS-CoV-2. A serological response to vaccination was achieved by 15 out of 21 (71.4%) patients in the JAKi cohort and 13 out of 16 (87.5%) patients in the TNFi cohort (*p* = 0.410). The median absolute anti-S IgG levels were 135.0 IgG BAU/ml (IQR 24.9–302.0) in the JAKi cohort and 129.0 IgG BAU/ml (IQR 54.1–418.5) in the TNFi cohort (*p* = 0.791) (Fig. 1D). Testing for a correlation between individual anti-S IgG levels and the frequency of specific IFNγ^+^/CD4^+^ T cells yielded a Spearman r of 0.01 (95% CI -0.032–0.34). Thus, the magnitude of humoral and cellular immune responses to vaccination were not related.

## Discussion

We compared the T cell response to SARS-CoV-S vaccination between patients taking JAKi and TNFi by performing ICS for IFNγ in CD4^+^ T cells after stimulation with SARS-CoV-2 spike peptides. While the T cell responsiveness to non-specific stimulation was similar in both groups, the strength of the vaccination-induced CD4^+^ T-cell response was significantly reduced in JAKi-treated patients compared to TNFi-treated patients. In contrast, the antibody response, as measured by anti-SARS CoV2 spike protein IgG, did not differ between JAKi- and TNFi- treated patients.

Our JAKi cohort had a significantly lower lymphocyte count than the TNFi cohort. Lymphopenia is known to be more common with JAKi therapy than with, for example, adalimumab [18]. However, the overall T lymphocyte function did not appear to be impaired, as the response to non-specific stimulation with PMA/ionomycin was not different between JAKi- and TNFi- treated patients.

Patient age, lymphopenia, concomitant medications, and the time since the last vaccination have been reported to correlate with a weak T cell response [19]. In our cohort, there was only an indication that lower lymphocyte counts were associated with a lower frequency of specific CD4^+^ T cells.

In previous studies T cell responses to SARS-CoV-2 vaccination were found to be generally weaker in immunosuppressed individuals compared to healthy controls. The percentage of patients with a specific T cell response, the frequency of specific T cells, and the high variability between subjects were similar to our findings [6, 7]. However, comparing ICS results is complicated by different methodologies. For instance, protocols with longer stimulation or co-stimulation may measure more cytokine-producing cells, but at the expense of lower specificity [20]. Our study’s finding of a significantly reduced T-cell response with JAKi is consistent with a study showing reduced T-cell responses under Tofacitinib after three doses of SARS-CoV-2 vaccination [4]. In contrast, other studies found no relevant differences in T-cell responses between patients on JAKi versus TNFi after two doses of vaccine [5–7].

There is limited evidence on cellular immune responses to vaccinations other than SARS-CoV-2 under JAKi therapy. It has been reported that the majority of patients showed specific CD4^+^ T cells after vaccination with recombinant zoster vaccine under upadacitinib and MTX. However, there was no comparison with a pre-vaccination sample to correct for pre-existing immunity in latent infection [21]. Another study showed satisfactory T cell–dependent antibody responses to polysaccharide conjugate pneumococcal vaccine (PCV-13) and tetanus vaccines in the majority of patients on tofacitinib therapy [22].

In our opinion, it is important to measure T cell responses to vaccination in immunosuppressed patients. In the context of SARS-CoV-2, this is underscored by the finding that specific T cells can persist for decades and provide long-lasting immunity when antibody levels are inadequate or have declined over time [23, 24]. In addition, T cells in vaccinated individuals can recognize viral variants that were not included in the original vaccine, which may provide some protection against upcoming new viral variants. However, it must be emphasised at this point that there is no exact correlate of T cell immunity following vaccination [25]. Therefore, measuring the percentage of circulating vaccine-induced, specific CD4^+^ T cells is a surrogate parameter of cellular immunity that does not necessarily indicate an immune response that protects against any kind of infection, as illustrated by breakthrough infections in SARS-CoV-2-vaccinated patients.

Our study’s limitations are its cross-sectional design and the small number of patients included and the variability in the period between vaccination and sample collection. The fact that half of the patients in the TNFi group had spondyloarthritis, while there were no patients with spondyloarthritis in the JAKi group, is a major confounder. Unfortunately, the number of patients included in the study was insufficient for a multivariate analysis on the influence of these potential confounding variables. The study aimed to investigate the de novo immune response in patients who had received two SARS-CoV-2 vaccinations and had no history of SARS-CoV-2 infection. However, due to the increase in infections and the requirement for booster vaccinations during the pandemic, the originally intended number of participants could not be achieved. For mRNA vaccines, it has been shown that the T cell responses elicited are mainly TH_1_ driven [13], which was investigated in our study by analysing CD4^+^ INFγ-production. Nevertheless, it would have been interesting to have a characterisation of the T-cell response extending to other cytokines, surface markers and CD8^+^ T cells.

## Conclusions

Our findings indicate a markedly impaired TH1 T cell response to SARS-CoV-2 vaccination in patients taking JAKi compared to TNFi. Our results emphasise the need for further investigation into the effect of JAKi on T cell responses. Based on these findings, strategies to improve the lasting success of vaccination in patients taking JAKi should be developed.

## Electronic supplementary material

Below is the link to the electronic supplementary material.

Supplementary Material 1

Supplementary Material 2

## Data Availability

The datasets used and/or analysed during the current study are available from the corresponding author on reasonable request.

## Abbreviations

ICS: Intracellular cytokine staining
anti-S IgG: Anti-spike IgG
IQR: Interquartile range
JAKi: Janus kinase inhibitors
PBMCs: Peripheral blood mononuclear cells
PMA: Phorbol-12-myristate-13-acetate
SD: Standard deviation
TNFi: Tumour necrosis factor-α-targeting biologics
