# Supplementary figures and images for: Comparison of T cell response to vaccination in rheumatic patients treated with Janus kinase inhibitors and TNF inhibitors
Source: BMC Rheumatol. 2025 Jul 9;9:84. doi: 10.1186/s41927-025-00542-7 (PMC12239352; doi:10.1186/s41927-025-00542-7)

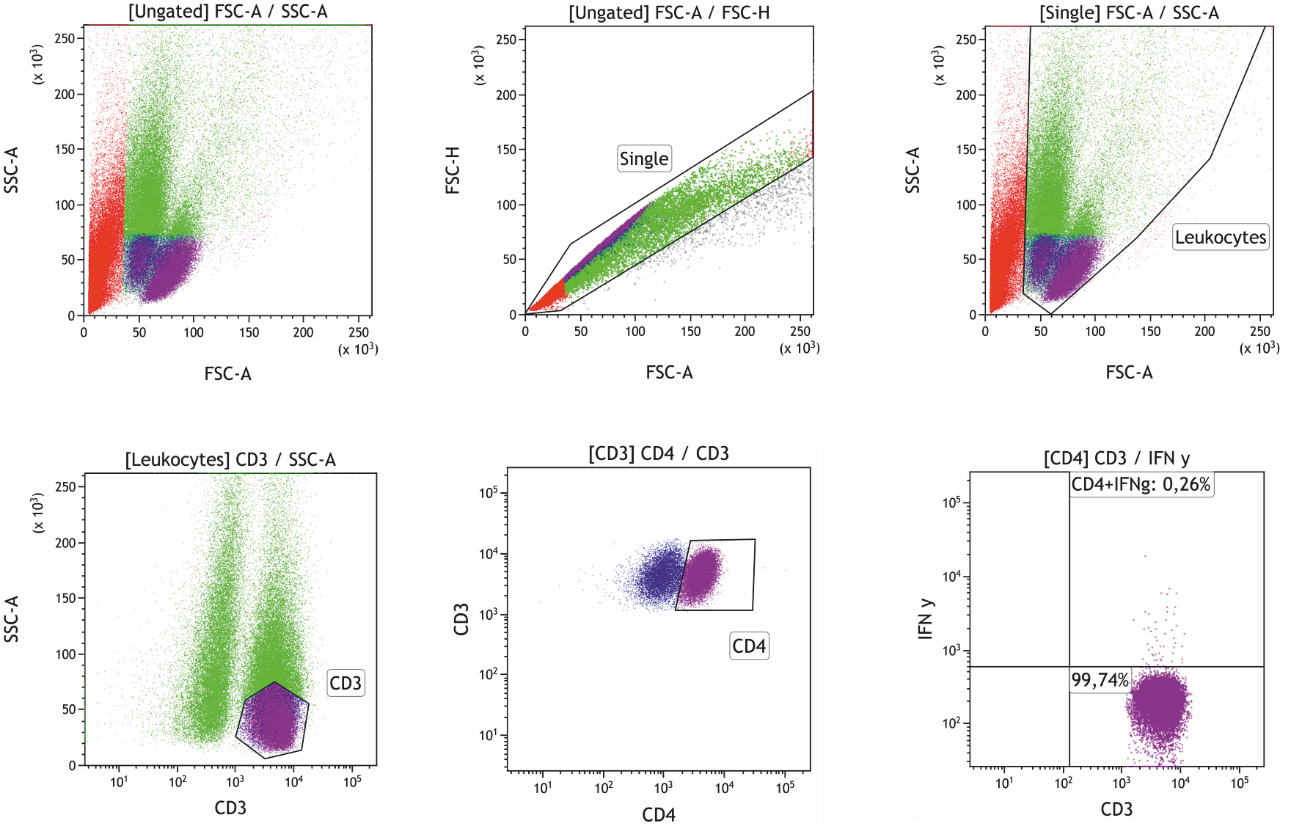


Supplementary Figure 1: Gating strategy for intracellular cytokine staining.

Supplement: Supplementary file 1 — Supplementary Material 1 [file 41927_2025_542_MOESM1_ESM.docx]
