# Supplementary material for: Comparison of T cell response to vaccination in rheumatic patients treated with Janus kinase inhibitors and TNF inhibitors
Source: BMC Rheumatol. 2025 Jul 9;9:84. doi: 10.1186/s41927-025-00542-7 (PMC12239352; doi:10.1186/s41927-025-00542-7)
Supplement: Supplementary file 2 — Supplementary Material 2 [file 41927_2025_542_MOESM2_ESM.docx]

Supplemental Table 1: Factors potentially affecting the T cell response to vaccination.

|  | **Spearman r(95% CI)** | **P value** |
| --- | --- | --- |
| Correlation between frequency of CD4^+^/IFNγ^+^ T cells and lymphocyte counts | 0.29 (-0.05 - 0.55) | 0.080 |
| Correlation between frequency of CD4^+^/IFNγ^+^ T cells and anti-S IgG titer | 0.01 (-0.32 - 0.34) | 0.946 |
| Correlation between frequency of CD4^+^/IFNγ^+^ T cells and patient`s age | 0.10 (-0.23 - 0.42) | 0.539 |
| Correlation between frequency of CD4^+^/IFNγ^+^ T cells and days since 2. vaccination | -0.05 (-0.37 - 0.29) | 0.785 |
| **Influence of immunosuppressive co-medication and discontinuation of medication during vaccination on the frequency of spike-specific CD4^+^ T cells:** | | |
| **Groups** | **Median frequency of CD4^+^/IFNγ^+^ T cells as % of all CD4^+^ (IQR)** | **P value** |
| Without MTX (n=23) | 0.010% (0.000 – 0.030%) | 0.152 |
| With MTX (n=15) | 0.020% (0.000 – 0.040%) |  |
| Without corticosteroids (n=30) | 0.015% (0.000 – 0.040%) | 0.594 |
| With corticosteroids (n=8) | 0.015% (0.000 – 0.028%) |  |
| Without pausing medication at vaccination (n=28) | 0.010% (0.000 – 0.038%) | 0.791 |
| With pausing medication at vaccination (n=10) | 0.020% (0.000 – 0.040%) |  |

The calculation was performed for all patients together. Correlations were tested using Spearman's rank correlation coefficient. The Mann-Whitney test was used to test for differences between groups.
